# Supplementary material for: Oases in the Sahara Desert–Linking biological and cultural diversity
Source: PLoS One. 2023 Aug 17;18(8):e0290304. doi: 10.1371/journal.pone.0290304 (PMC10434913; doi:10.1371/journal.pone.0290304)
Supplement: S1 Fig — Pearson correlation (r) and significance (p) are shown. (DOCX) [file pone.0290304.s001.docx]

**Supplementary Material 2**

**Oases in the Sahara Desert – Linking Biological and Cultural Diversity**


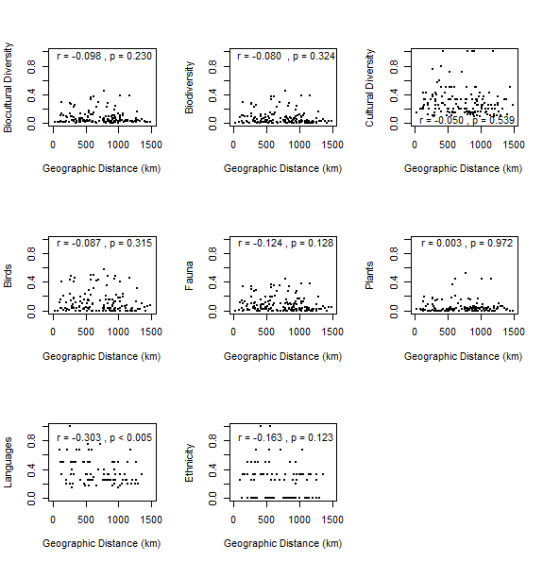


**Figure S1**: Relationship between biological and cultural diversity and geographic distances in oases groups in Algeria. Pearson correlation (*r*) and significance (*p*) are shown
