# Supplementary material for: Oases in the Sahara Desert–Linking biological and cultural diversity
Source: PLoS One. 2023 Aug 17;18(8):e0290304. doi: 10.1371/journal.pone.0290304 (PMC10434913; doi:10.1371/journal.pone.0290304)
Supplement: Attachment — Submitted filename: Response letter_21_07.docx [file pone.0290304.s003.docx]

---------------------------------------------------------------------------------------------------

**Response to Academic Editor’s comments**

1. Please ensure that your manuscript meets PLOS ONE's style requirements, including those for file naming. The PLOS ONE style templates can be found at

https://journals.plos.org/plosone/s/file?id=wjVg/PLOSOne_formatting_sample_main_body.pdf and

https://journals.plos.org/plosone/s/file?id=ba62/PLOSOne_formatting_sample_title_authors_affiliations.pdf

R: *The manuscript has been revised and format requirements are met.*

2. Thank you for stating the following financial disclosure:

"Deutsche Forschungsgemeinschaft (DFG; JE 288/9-1, 9-2)"

Please state what role the funders took in the study. If the funders had no role, please state: "The funders had no role in study design, data collection and analysis, decision to publish, or preparation of the manuscript."

If this statement is not correct you must amend it as needed.

Please include this amended Role of Funder statement in your cover letter; we will change the online submission form on your behalf.

R: *A Financial Disclosure Statement has been included with information about the role of funders in the study.*

3. Thank you for stating the following in the Acknowledgments Section of your manuscript:

"We thank Joanna Mitchell and Merve Ören for collecting data on oasis settlements in the Sahara Desert. JMJ acknowledges financial support from the Deutsche Forschungsgemeinschaft (DFG; JE 288/9-1, 9-2)."

We note that you have provided funding information that is not currently declared in your Funding Statement. However, funding information should not appear in the Acknowledgments section or other areas of your manuscript. We will only publish funding information present in the Funding Statement section of the online submission form.

Please remove any funding-related text from the manuscript and let us know how you would like to update your Funding Statement. Currently, your Funding Statement reads as follows:

"Deutsche Forschungsgemeinschaft (DFG; JE 288/9-1, 9-2)"

Please include your amended statements within your cover letter; we will change the online submission form on your behalf.

R: *A Financial Disclosure Statement has been included with information about the funding including the specific grant number, initials of the author who received the award and the URL of the sponsor’s website.* *In the acknowledgement statement we added the following sentence:*

*L572-573: “**We are very grateful to two reviewers and the Editor for providing very helpful comments that considerably improved the text.”*

4. Please note that in order to use the direct billing option the corresponding author must be affiliated with the chosen institute. Please either amend your manuscript to change the affiliation or corresponding author, or email us at plosone@plos.org with a request to remove this option.

*R: Please use Senckenberg as first affiliation and add the billing address: Senckenberganlage 25, 60325 Frankfurt, Germany; Email: generaldirektion@senckenberg.de*

5. We note that Figure 1 in your submission contain [map/satellite] images which may be copyrighted. All PLOS content is published under the Creative Commons Attribution License (CC BY 4.0), which means that the manuscript, images, and Supporting Information files will be freely available online, and any third party is permitted to access, download, copy, distribute, and use these materials in any way, even commercially, with proper attribution. For these reasons, we cannot publish previously copyrighted maps or satellite images created using proprietary data, such as Google software (Google Maps, Street View, and Earth). For more information, see our copyright guidelines: http://journals.plos.org/plosone/s/licenses-and-copyright.

We require you to either (1) present written permission from the copyright holder to publish these figures specifically under the CC BY 4.0 license, or (2) remove the figures from your submission:

a. You may seek permission from the original copyright holder of Figure 1 to publish the content specifically under the CC BY 4.0 license.

We recommend that you contact the original copyright holder with the Content Permission Form (http://journals.plos.org/plosone/s/file?id=7c09/content-permission-form.pdf) and the following text:

“I request permission for the open-access journal PLOS ONE to publish XXX under the Creative Commons Attribution License (CCAL) CC BY 4.0 (http://creativecommons.org/licenses/by/4.0/). Please be aware that this license allows unrestricted use and distribution, even commercially, by third parties. Please reply and provide explicit written permission to publish XXX under a CC BY license and complete the attached form.”

Please upload the completed Content Permission Form or other proof of granted permissions as an "Other" file with your submission.

In the figure caption of the copyrighted figure, please include the following text: “Reprinted from [ref] under a CC BY license, with permission from [name of publisher], original copyright [original copyright year].”

b. If you are unable to obtain permission from the original copyright holder to publish these figures under the CC BY 4.0 license or if the copyright holder’s requirements are incompatible with the CC BY 4.0 license, please either i) remove the figure or ii) supply a replacement figure that complies with the CC BY 4.0 license. Please check copyright information on all replacement figures and update the figure caption with source information. If applicable, please specify in the figure caption text when a figure is similar but not identical to the original image and is therefore for illustrative purposes only.

The following resources for replacing copyrighted map figures may be helpful:

USGS National Map Viewer (public domain): http://viewer.nationalmap.gov/viewer/

The Gateway to Astronaut Photography of Earth (public domain): http://eol.jsc.nasa.gov/sseop/clickmap/

Maps at the CIA (public domain): https://www.cia.gov/library/publications/the-world-factbook/index.html and https://www.cia.gov/library/publications/cia-maps-publications/index.html

NASA Earth Observatory (public domain): http://earthobservatory.nasa.gov/

Landsat: http://landsat.visibleearth.nasa.gov/

USGS EROS (Earth Resources Observatory and Science (EROS) Center) (public domain): http://eros.usgs.gov/#

Natural Earth (public domain): <http://www.naturalearthdata.com/>

*R: The map of Figure 1 does not contain images with copyright. We created the map ourselves based on our results. The land outline is taken from Natural Earth (public domain). We included information on trade route nodes from the Old World Trade Routes Project. While all materials on trade routes on the website are published under a Creative Commons BY NC 2.5 license (http://creativecommons.org/licenses/by-nc/2.5/), this license does not apply to the raw data published in the Old World Trade Routes (OWTRAD) Gazetteer (http://www.ciolek.com/OWTRAD/gazetteer-01.html), which is not copyrighted. We have included the trade node points based on the raw coordinates from the Gazetteer, therefore this image can be published under CC BY 4.0. To ensure we cite all the information provided, we added a citation for this information:*

*L201-202: “based on Old World Trade Routes Project: http://www.ciolek.com/owtrad.html [60]”*

*And*

*L734-735: “Ciolek TM. Old World Trade Routes (OWTRAD) Project. 1999* [*http://www.ciolek.com/owtrad.html*](http://www.ciolek.com/owtrad.html)*”*

6. Please remove your figures from within your manuscript file, leaving only the individual TIFF/EPS image files, uploaded separately. These will be automatically included in the reviewers’ PDF.

*R: We have removed the figures from the manuscript.*

7. Please include captions for your Supporting Information files at the end of your manuscript, and update any in-text citations to match accordingly. Please see our Supporting Information guidelines for more information: http://journals.plos.org/plosone/s/supporting-information.

*R: We have included captions for the Figure S1 at the end of the document:*

*L841-842 Figure S1: “Relationship between biological and cultural diversity and geographic distances in oases groups in Algeria. Pearson coefficients (r) and significance levels (p) are shown”.*

8. Please review your reference list to ensure that it is complete and correct. If you have cited papers that have been retracted, please include the rationale for doing so in the manuscript text, or remove these references and replace them with relevant current references. Any changes to the reference list should be mentioned in the rebuttal letter that accompanies your revised manuscript. If you need to cite a retracted article, indicate the article’s retracted status in the References list and also include a citation and full reference for the retraction notice.

*R: All references have been reformatted and double-checked.*

-----------------------------------------------------------------------------------------------------

**Response to reviewers’ comments**

**Reviewer 1’s comments**

Reviewer #1: This is an interesting, well-written and well-structured paper dealing with the biocultural diversity approach which focuses on the co-evolution of biological and cultural systems, using oases in the Sahara Desert as model systems. This work discusses potential drivers and proxies of changing biological and cultural diversity starting from a well-reasoned conceptual framework, and then investigates the biocultural diversity of Algerian oases by applying two complementary methods testing the influence of area and human population.

Linking biological and cultural diversity is a challenging topic especially because of the difficulty of exploring patterns and dynamics of cultural diversity. Nevertheless, this kind of approach is of paramount importance and deserves great attention due to the ongoing globalization processes and environmental degradation that are threatening the biocultural diversity across the world.

The Authors addressed the issue of quantifying biocultural diversity by testing existing (and adapted) indices on data of Sahara oases gathered from an extensive literature review. Overall the results are compelling and indicate that the method is suitable for assessing the biocultural diversity in the Algerian oases. My main concern is that the literature data are not homogeneous for each oasis and therefore some proxy is not properly ‘weighted’ (e.g., in which way the results are influenced by poor data as in the case of oasis Abelassa in the Hoggar group?). I suggest adding a couple of sentences explaining in which way the proposed method overcomes such flaws.

*R: We are grateful to the reviewer for the positive and encouraging assessment of the manuscript. However, the review identifies one limitation of the study, and we addressed this by including the following statement:
L482-491: “**A key challenge of the present study was the lack of qualified data and information for the oases in the Sahara Desert. Consequently, we could only extract parts of our ‘proxy wish list’ presented above. In addition, JI considers only pairwise beta diversity and does not provide information on the underlying phenomena such as nestedness (i.e., oases with smaller numbers of species are subsets of oases with higher numbers of species) and turnover (replacement of species by other species). Hence, multiple-site comparisons, including nestedness and turnover, are considered important for future studies [84,92].* *In addition, JI is not adjusted for area, which might cause biased results [92].* *Lastly, we lack comprehensive data and information to understand and quantify the temporal dynamics of biocultural diversity in the Sahara desert.”*

As regards to the conceptual framework, the Authors have the merit of exposing in a clear and scientific way the potential drivers of biological and cultural diversity. Nevertheless, in my opinion the temporal scale – which has an essential role in patterns and trends of biocultural diversity – is not properly introduced. I recommend better explaining why such a ‘deletion’ of a diachronic perspective might matter.

*R: As mentioned above, we could not address the temporal dynamics in our analyses (L326-327 “Here, we provide an analysis of contemporary patterns of Algerian oases, ignoring temporal trends”) due to a lack of comprehensive data and information of those understudied ecosystems. We have added an explanation for this:*

*L489-491: “**Lastly, we lack comprehensive data and information to understand and quantify the temporal dynamics of biocultural diversity in the Sahara desert.”*

Also, the cited references in the section 3 ‘Linking biological and cultural diversity in oases’ should be improved (e.g., in the par. 3.1.4 the Authors cannot cite only Kuper & Kröpelin 2006 as reference for the settlement history of the Saharan area!). I recommend widen the reference literature of the conceptual framework, so that the well-written text may be improved with relevant and updated reference literature.

*R: Additional relevant references such as Manning et al. (2013), Marshall & Weissbord (2011) or Manning & Timpson (2014) have been included in the text.*

The drawing of the follow-up research questions is one of the strong points of the manuscript.

Overall, the topic is in line with the journal and my advice is that the paper needs minor revisions before publication.

**Reviewer 2’s comments**

Reviewer #2: Dear authors,

your paper is interesting and well structured. THe addressed topic is particulalry relevant for the conservation of oases-related cultural biodiversity.

I only suggest some minor revisions.

The introduction provide a good framework about the issue addressed in the paper, but it would be also interesting to highlight the difference between traditional and modern oases, to clarify the focus of your research.

*R: We are very grateful for the positive assessment of our manuscript and the helpful comments. We have added a sentence mentioning traditional oases at the end of the introduction:*

*L74-76: “forming pivotal stepping stones along trade routes and supporting social and economic innovations [27], specially, especially traditional oases [31]”*

*And in objectives:*

*L87-88: “…using traditional oases (sensu [31]) in the Sahara Desert as model systems”*

Lines 76-85. I suggest authors to expand this paragraph with more recent references (the more recent is of 2012) as there are interesting updated studies about oases and related ecosystem services and agro-biodiversity. I.e. studies highlighting that oases far from market centers (as Lybian ones) preserve a higher level of agrobiodiversity.

*R: We have included more recent references to the paragraph such as Berger et al., 2021 Santoro, 2023 and Santoro et al., 2020.*

Lines 265-266. I suggest to include the FAO definition of agrobiodiversity, or at least its citation.

*R: We have added the reference FAO, 1999 to the text:*

*L262: “…particularly for agrobiodiversity [69–71]”.*

Line 281, not only species, but also varieties, especially if you refer to date palm (1 species, but a lot of varieties!)

*R: We have included varieties in the text:*

*L277-278 “Hence, the number of different cattle and crop species and varieties should be identified for oases”*

Line 530-531. This can be true, but if you state "at present" you should add a "present" reference. Since 2018 (the most updated reference) socio-economic situation could be really different.

*R: We rephrased this as:*

*L530-531 : “**Unstable political conditions (e.g., rebel activities) constrain research in many oases of the Sahara Desert [89,101].*
